# Effect of Divalent Metal Ions on the Ribonuclease Activity of the Toxin Molecule HP0894 from *Helicobacter pylori*

**DOI:** 10.3390/life14020225

**Published:** 2024-02-05

**Authors:** Ja-Shil Hyun, Rabin Pun, Sung Jean Park, Bong-Jin Lee

**Affiliations:** 1 Gachon Institute of Pharmaceutical Sciences, College of Pharmacy, Gachon University, 191 Hambakmoero, Yeonsu-gu, Incheon 21936, Republic of Korea; 2 College of Pharmacy, Ajou University, 206 World Cup-ro, Yeongtong-gu, Suwon 16499, Republic of Korea

**Keywords:** toxin–antitoxin system, *Helicobacter pylori*, Ribonuclease, HP0894, zinc

## Abstract

Bacteria and archaea respond and adapt to environmental stress conditions by modulating the toxin–antitoxin (TA) system for survival. Within the bacterium *Helicobacter pylori*, the protein HP0894 is a key player in the HP0894-HP0895 TA system, in which HP0894 serves as a toxin and HP0895 as an antitoxin. HP0894 has intrinsic ribonuclease (RNase) activity that regulates gene expression and translation, significantly influencing bacterial physiology and survival. This activity is influenced by the presence of metal ions such as Mg^2+^. In this study, we explore the metal-dependent RNase activity of HP0894. Surprisingly, all tested metal ions lead to a reduction in RNase activity, with zinc ions (Zn^2+^) causing the most significant decrease. The secondary structure of HP0894 remained largely unaffected by Zn^2+^ binding, whereas structural rigidity was notably increased, as revealed using CD analysis. NMR characterized the Zn^2+^ binding, implicating numerous His, Asp, and Glu residues in HP0894. In summary, these results suggest that metal ions play a regulatory role in the RNase activity of HP0894, contributing to maintaining the toxin molecule in an inactive state under normal conditions.

## 1. Introduction

Bacteria and archaea respond and adapt to environmental stress by modulating the toxin–antitoxin (TA) system for survival [1,2,3]. The TA system consists of a stable toxin that can harm cells and a labile antitoxin that neutralizes the toxin under normal physiological conditions. The antitoxins can be either proteins or RNAs depending on type of the TA systems. However, under stress conditions, such as nutrient deprivation or a rise in temperature, specific proteases are triggered and rapidly degrade unstable or labile antitoxins. The imbalance between toxin and antitoxin concentrations causes free toxins to interact with cellular processes, inhibiting cell growth, and leading to cell death [3,4,5,6]. Depending on their functions, TA systems can be classified into eight types [7,8,9]. In type I TA systems, short peptides containing a single helix are the majority of type I toxins, exemplified by Hok and Fst that are membrane-associated peptides that disrupt the membrane [7]. The unusual type I toxin SymE is larger than most of the peptide toxins and is a nucleoid-associated protein [8]. The antisense RNA, which acts as an antitoxin, interrupts the translation of the toxin mRNA [10,11,12]. Type II TA systems are relatively well known. Most toxin molecules in the system are codon-dependent endoribonucleases, and antitoxin proteins form stable complexes with toxin proteins, thereby playing a role in plasmid maintenance [12,13]. Examples of Type II include RelB-RelE, DinJ-YafQ, and YefM-YoeB pairs [1]. *Helicobacter pylori* contains four pairs of Type II TA systems: HP0315-HP0316 [14], HP0892-HP0893 [15], HP0894-HP0895 [16], and HP0967-HP0968 [17]. In Type III TA systems, the interaction of RNA molecules, such as the YafN–YafO pair, inhibits toxin activity [10]. This type contains RNA antitoxins that bind to and neutralize toxin proteins [12,18]. The remaining types are explained elsewhere [9,19]. The *Escherichia coli* K-12 chromosome codes for at least 12 different type II TA systems, including relBE, yefM–yoeB, mazEF, dinJ–yafQ, hipBA, chpBK, and mqsAR [7,9,20]. Most type II toxins exhibit mRNase activity with different specificities, whereas some affect replication by interacting with DNA gyrase (CcdB families) and inhibiting peptidoglycan biosynthesis (ζ-toxins) [9,21,22].

It has been estimated that one-fourth to one-third of all proteins rely on metal ions, although in varying quantities [23,24]. Many RNases exhibit a dependence on divalent metal ions for their activity. These ions play crucial roles in the catalytic mechanism by producing reactive water molecules, stabilizing the transition state, and aiding in the correct positioning of the RNA molecule at the enzyme active site [25]. Given that toxin molecules in TA systems often function as RNases, our investigation focused on whether these toxin molecules interact with specific divalent metal ions. These interactions between RNases and metal ions are complicated and do not follow a typical mechanism. Some RNases exhibit activity in the presence of divalent metal ions, whereas others function in the presence of metal cheaters such as EDTA [26]. For instance, *E. coli* RNase H has a binding site for Mg^2+^, and Mg^2+^ or Mn^2+^ serve as a cofactor during catalysis in *Bacillus halodurans* [27]. In contrast, Ca^2+^ ions inhibit RNase H catalysis in *B. halodurans* [28], and RNase T in *E. coli* is activated by Ca^2+^ ions [29]. Additionally, Mg^2+^ binds to the active site of RNase II in Rrp44 [30].

In previous studies we elucidated the structure and function of two Type II TA systems from *H. pylori*, namely HP0892-HP0893 and HP0894-HP0895. In both systems, HP0892 and HP0894 act as toxins that form complexes with the antitoxins HP0893 and HP0895, respectively [15,16,20,31]. HP0892 is a homologue of HP0894 with 54% amino acid identity. HP0894 is a short protein composed of 88 amino acids. Its structure contains four α-helices and four β-strands, providing a platform for substrate binding and catalysis. The β-sheet consisting of four β-strands is responsible for ribonuclease activity, allowing it to cleave RNA molecules [21]. The conserved amino acid residues such as His47, His60, and His84 are important for catalytic activity. HP0894-like toxins are conserved among several species of *Helicobacter* with similar lengths (Supplementary Figure S1). This system is found in 218 out of thousands complete genomes of *H. pylori* strains (Supplementary Figure S2).

However, the characteristics of the interactions between HP8094 and divalent metals remain unknown, with the exception of the Cu^2+^ bound structure [20]. Therefore, we investigated the interactions between HP0894 and various divalent metal ions. This study revealed that, among the various divalent metal ions examined, Zn^2+^ significantly suppressed the RNase activity of HP0894, resulting in a diminished cleavage of the target mRNA. The secondary structure of HP0894 remained largely unaffected by Zn^2+^ binding, whereas its structural rigidity markedly increased due to Zn^2+^. Furthermore, through NMR analysis, we identified the residue-specific Zn^2+^ binding site.

## 2. Materials and Methods

### 2.1. Preparation of Protein Samples

HP0894 was amplified using *H. pylori* (strain ATCC 700392/26695) genomic DNA (Uniprot ID: O25554_HELPY/Genbank ID: AAD07942) as a template. The PCR products were digested with *Nco*I and *Nde*I restriction enzymes (NEB, Ipswich, MA, USA) and ligated into a pre-digested pET-15b vector (Novagen Inc. Madison, WI, USA). The construct harbored an N-terminal hexahistidine tag and thrombin cleavage site to facilitate purification. Overexpression of the recombinant plasmid was achieved through transformation into *E. coli* BL21(DE3) competent cells (Novagen, Inc.). Protein overexpression and purification were performed as described previously [20]. The protein was purified using an open Ni^2+^-NTA column (Qiagen, Germantown, MD, USA), and the non-native hexahistidine tag was cleaved off with thrombin digestion at 20 °C for 12 h. Thrombin cleavage resulted in three non-native residues (GSH) attached to the N-terminus of HP0894. The protein was then subjected to gel filtration (Superdex 75 10/300 GL; GE Healthcare Sciences, Seoul, Republic of Korea) to achieve further purity [32].

To prepare labeled 15N-HP0894 for NMR experiments, *E. coli* BL21(DE3) cells containing the recombinant plasmid were grown in M9 medium with uniformly labeled 15N ammonium chloride [U-15N] (Cambridge Isotope Laboratories Inc., Tewksbury, MA, USA) as the sole nitrogen source. The remainder of the process for protein isolation and purification was the same as described above. Approximately 150 μM of 15N labeled HP0894 was prepared in 20 mM MES buffer (pH 6.0) containing 100 mM NaCl and 10% (*v*/*v*) D_2_O.

### 2.2. The Ribonuclease Activity (RNase) Activity of HP0894 and Interaction of Divalent Metal Ions

The RNase activity of HP0894 was assessed using a fluorometric method, in which 10-base-long mRNA (5′-ACACUAAGAA-3) was chosen as a substrate [33]. A fluorophore (6-FAM) was covalently labeled to one end of the mRNA substrate and quenched by a quencher group (BHQ) attached to the other end of the mRNA substrate. If the RNase processes synthetic mRNA that harbors a fluorophore-quencher pair, the separation of the fluorophore and quencher pair results in an increase in fluorescence at 520 nm (excitation at 490 nm). The fluorescence intensities were measured, and the curves were obtained by subtracting the buffer signals. The mRNA substrates (Bioneer, Daejeon, Republic of Korea) were synthesized commercially.

Mixtures of HP0894 and various divalent metal ions were prepared at appropriate ratios and incubated at 37 °C for 1 h. To clarify the absence of metals, we pre-treated the protein sample with 2 mM EDTA and then dialyzed the protein solution to remove remaining EDTA. A final concentration of 8 μM of HP0894 was added to each fluorescent substrate tube using 20 mM MES buffer (pH 6.0) containing 100 mM NaCl, and we employed a total reaction volume of 50 μL. The reactions were carried out in the absence (apo-HP0894) or presence of 2 mM of each metal ion (Cu^2+^, Ca^2+^, Mn^2+^, Mg^2+^, Co^2+^, Ni^2+^ or Zn^2+^). The samples were loaded into a 384-well plate, and we measured fluorescence (excitation 490 nm/emission 520 nm) using the Gemini XPS Microplate Reader instrument (Molecular Devices, San Jose, CA, USA) with readings taken every 1 min for 3 h 30 min at 37 °C.

### 2.3. Circular Dichroism Spectroscopy (CD)

Measurements for CD were carried out in 20 mM sodium phosphate buffer (pH 5.0) containing 100 mM NaCl using a 2 mm path length quartz cuvette (Starna Cells Inc., Atascadero, CA, USA) in a J-715 spectropolarimeter (JASCO Corporation, Tokyo, Japan) equipped with a Peltier temperature-control system (Model PTC-348WI). All samples were scanned with a bandwidth of 1 nm and a response time of 0.5 s at a scanning speed of 50 nm/min. All spectra were recorded as the mean of three scans. To monitor the metal effects, the metal mixtures in which 20 μM of HP0894 was mixed with 30 μM of different metal ions was prepared or the zinc mixtures in which 20 μM of HP0894 was mixed with various concentration of zinc ions (Zn^2+^). The signals were monitored in Far-UV region (190–250 nm). We also conducted near-UV CD measurements (250–350 nm) using 200 μM HP0894 in the apo form with the gradual addition of Zn^2+^. Data were processed with blank subtraction and smoothed before analysis. The change in molar ellipticity [θ] was calculated using the following equation [34], where θ is in millidegrees, pathlength (l) is in millimeters, and C is the molar concentration of the protein:

[θ] = θ/(l × C × number of residues)

The thermal unfolding of HP0894 was examined upon the addition of metal ions, and the temperature scanning was conducted with a constant 1 °C increase from 20 °C to 90 °C at 229 nm. A bandwidth of 2 nm and a response time of 4 s were employed. The data were normalized and smoothed before analysis, and the melting temperature data were normalized using Origin 5 Software (OriginLab, Northampton, MA, USA).

### 2.4. Isothermal Titration Calorimetry (ITC) Assay

Proteins were dialyzed extensively in 20 mM MES buffer (pH 6.0) containing 100 mM NaCl. The ZnCl_2_ was dissolved in the same buffer solution. Both the protein and ZnCl_2_ were degassed and filtered before use, and isothermal titration calorimetry measurements were carried out using a VP-ITC instrument (MicroCal, GE Healthcare Sciences, Seoul, Republic of Korea). The protein was concentrated to 100 μM and added to the experimental chamber, whereas the injection syringe was filled with 1.5 mM zinc chloride. The experiment consisted of 25 injections with an initial delay of 60 s with a 10 μL initial injection volume, followed by a 20 s delay, and an injection volume of 12 μL followed by a 300 s delay for the remainder of the injections. A stirring speed of 1000 rpm at 25 °C (±0.1 °C) was employed. Origin software was used to integrate the heat signals obtained from the raw ITC data, and the background heat from the reference buffer was subtracted to obtain the corrected heat. A single-site binding isotherm model was used for data fitting and the binding affinity (K), change in enthalpy (ΔH), change in entropy (ΔS), and binding stoichiometry (N) were calculated.

### 2.5. Nuclear Magnetic Resonance (NMR) Spectroscopy

A series of 2D-[1H-15N] HSQC spectrum for 150 μM of 15N-labeled apo-HP0894 was obtained through the addition of the increasing concentration of zinc in 1:1, 1:1.5, 1:2, 1:2.5, and 1:3 ratios on Bruker Avance III 600 MHz spectrophotometer (Bruker Korea Co., Seongnam, Republic of Korea) at 30 °C [35]. The chemical shifts were externally referenced to DSS. The δ ppm values for the backbone N and HN resonances of HP0894 were assigned from data obtained in an earlier study (PDB code 1Z8M) [16]. Chemical shift perturbation experiments were performed by titrating HP0894 with Zn^2+^ and the disappearance and/or shifting of peaks were monitored. All NMR spectra were processed using NMRPipe and nmrDraw [36] and analyzed using NMRView [37]. The average chemical shift change was calculated using the following equation [38]:

Δδ_ave_ = [(Δδ_HN_)^2^ + (Δδ_N_/6.57)^2^]^0.5^

## 3. Results

### 3.1. HP0894 Ribonuclease (RNase) Activity of HP0894 and Interference of Divalent Metal Ions

**Figure 1:**
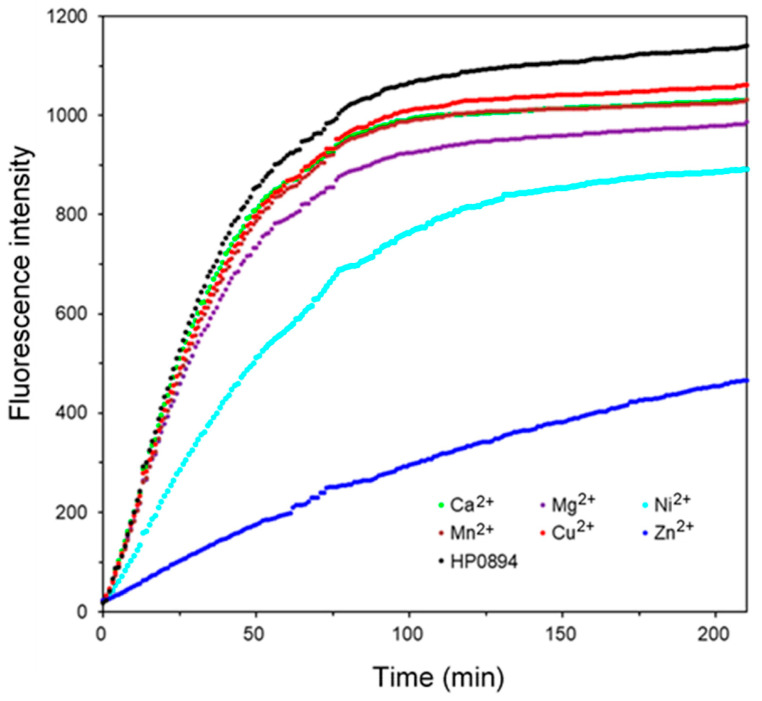
HP0894 RNase inhibition test. The amount of ribonuclease (RNase) activity exerted by HP0894 for 3 h 30 min at 37 °C, and the inhibitory effect of different metal ions on the activity was monitored.

The RNase activity of HP0894 and the effects of different metal ions were observed. Intrinsically, HP0894 exhibited mild RNase activity compared to other RNases, as observed in Figure 1. Interestingly, all metal ions caused a slight or significant reduction in the RNase activity of HP0894. Half of the substrate RNA was degraded within 25 min in the presence of Ca^2+^, Cu^2+^, Mn^2+^, or Mg^2+^, whereas Ni^2+^-bound HP0894 exhibited an extended half-degradation time. Most importantly, the addition of Zn^2+^(blue) resulted in a three-fold decrease in the fluorescence emission compared to that of the apo form, and reaction saturation was not achieved within the experimental time. Thus, these results indicated that the RNase activity of HP0894 was highly inhibited by Zn^2+^.

### 3.2. Effect of Metal Ions on the Secondary Structure of HP0894

**Figure 2:**
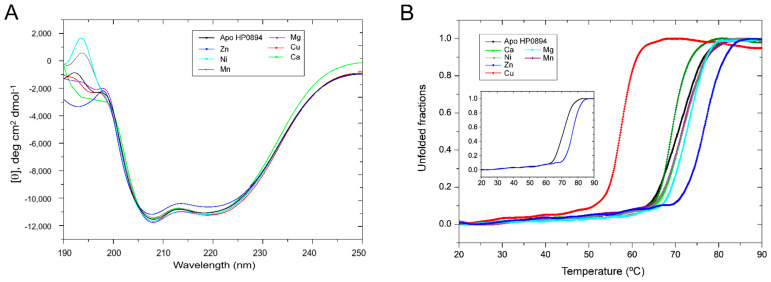
Secondary structural analysis and thermal unfolding studies with circular dichroism. (**A**) Effect of different metal ions on the secondary structure of HP0894. In total, 20 μM HP0894 was mixed with 30 μM metal ions, and the observed spectra are shown. No significant change in the secondary structure was observed. (**B**) Thermal denaturation of HP0894 was carried out and the unfolded fractions were plotted against the increase in temperature to observe the effect of metal ions on the stability of HP0894. Tm of apo-HP0894 was observed at 72 °C (black) and at 77 °C upon addition of zinc (blue). The inset illustrates the Tm of apo- and zinc-bound HP0894 for clarity.

We measured CD spectra in the far-UV region (190–250 nm) to observe the effects of different metal ions on the structure of HP0894. The 20 μM apo-HP0894 exhibited the characteristic minima at 209 nm and 222 nm, indicating α-helical structure of the protein, and the effect of addition of different metal ions was observed (Figure 2A). Except for zinc, the pattern of the CD spectra upon the addition of metal ions did not exhibit a change compared with the spectrum of apo-HP0894. The helical propensity of apo-HP0894 was around 54%, which was calculated using the CDNN program (Applied Photophysics Ltd., Leatherhead, UK), while the zinc-bound HP0894 exhibited 46% of α-helicity in the structure. To determine the effect of these metal ions on the stability of the HP0894 protein, thermal denaturation experiments were carried out, and the differences in melting temperature (Tm) were compared. A plot of the intensity change at 222 nm with increasing temperature is represented in Figure 2B. The apo-HP0894 (black) displayed Tm at 72 °C, and Tm alteration in the presence of Ca^2+^, Mn^2+^, and Mg^2+^ was varied within 2 °C from 72 °C. The maximum change of Tm was observed in zinc-bound HP0894 (blue) at 77.5 °C, which may suggest the structural rigidity of HP0894 was highly increased with zinc binding. Interestingly, the heat stability of HP0894 was reduced in the presence of Cu^2+^ (Figure 2B), while the overall secondary structure was not significantly changed with Cu^2+^ as observed in Figure 2A. Cu binding appears to destabilize HP0894.

**Figure 3:**
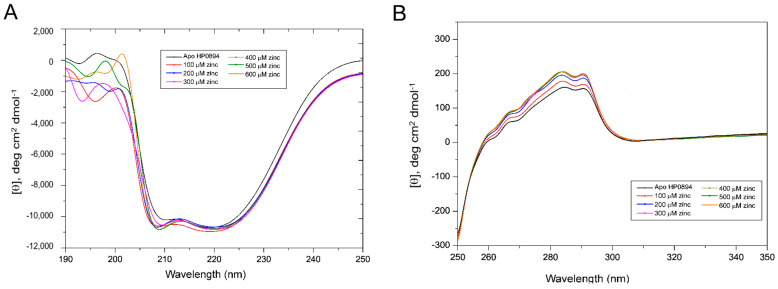
Effect of zinc on HP0894. (**A**) Effect of zinc ions on the secondary structure of HP0894. We titrated 20 μM HP0894 against increasing concentration of zinc ions, and the change in molar ellipticity [θ] was observed. (**B**) Near-UV CD spectrum. Near UV-CD spectra of HP0894 upon addition of increasing concentration of zinc ions. The change in molar ellipticity [θ] was observed at 284 nm and 291 nm.

To further investigate the effect of Zn^2+^ on HP0894, change in molar ellipticity [θ] upon increasing concentration of Zn^2+^ was monitored. Though the Zn^2+^ did not exhibit significant change in the far-UV region (Figure 3A), the intensification of molar ellipticity [θ] at 208 nm was observed. This suggests that the helical structure of HP0894 is stabilized with Zn^2+^ binding. Notable change of CD signals was observed in the near-UV region according to the addition of zinc (Figure 3B): concentration-dependent increase in molar ellipticity [θ] was observed at 284 nm and 291 nm, indicating that zinc binding affects the conformation state of aromatic residues (especially for His and Phe that are major aromatic residues in HP0894). Overall, Zn^2+^ and other metal ions, except Cu, stabilized the secondary structures and rigidity of the tertiary fold of HP0894. In addition, the conformation of aromatic residues may be affected by Zn^2+^, indicating that the coordination of Zn^2+^ with these residues may be important for structural stabilization (Figure 3B).

### 3.3. Thermal Analysis and Binding Stoichiometry Using Isothermal Titration Calorimetry

**Figure 4:**
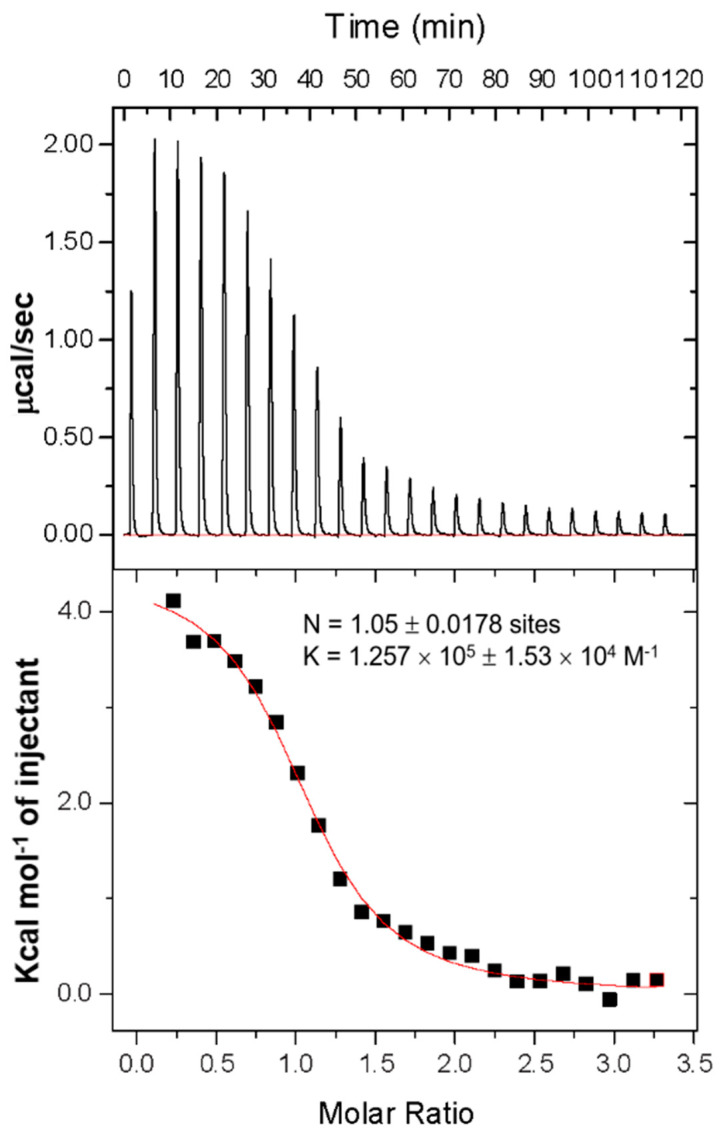
ITC analysis of zinc bound HP0894. Top panel: total heat change upon addition of zinc chloride into the chamber cell containing HP0894. Bottom panel: area underneath each peak is integrated (black square). A single-site binding isotherm model was used to fit data (red line), and the resulting calculations for binding stoichiometry (N) and the binding affinity (K) are illustrated.

To further characterize the Zn^2+^ effect, ITC was used to determine the thermodynamic nature of the interaction and the exact binding stoichiometry between HP0894 and Zn^2+^ (Figure 4). The binding of zinc to HP0894 resulted in an endothermic reaction. Based on the ITC results, HP0894 contains a single binding site for Zn^2+^, and their dissociation constant was approximately 8 μM. Previously, we evidenced that HP0894 can be coordinated by Cu^2+^ through His47, Glu58, His60, and His84 of HP0894 with 0.5:1 (Cu^2+^:HP0894) stoichiometry [20], whereas the zinc ion may bind to HP0894 with 1:1 stoichiometry. The binding enthalpy and entropy derived from ITC data were 8246 ± 54 cal/mol and 51 cal/mol/deg, respectively, which indicates that Zn^2+^ binding is an entropy-driven interaction.

### 3.4. Zinc Titration and NMR Perturbation Experiments

**Figure 5:**
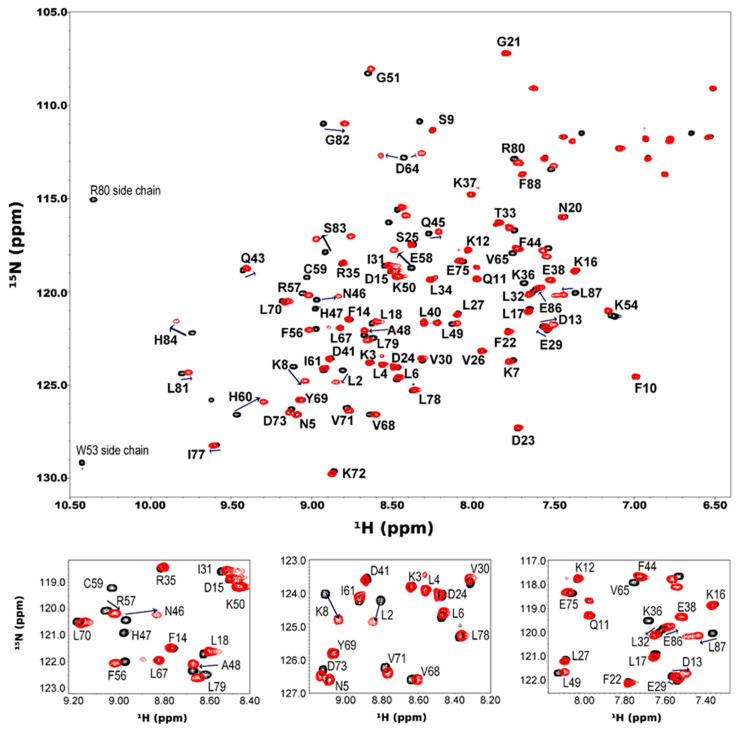
NMR titration of HP0894 with zinc. A series of two-dimensional [1H-15N] HSQC spectra of 150 μM U-15N HP0894 alone (black) and upon addition of 300 μM ZnCl2 (red) are illustrated. The overlapping regions in the HSQC spectra are illustrated in the inset for clarity, as well as the side chain of aromatic amino acids. The arrows represent the peaks shifting.

A series of 2D-[1H-15N] HSQC spectra of HP0894 was acquired upon the addition of ZnCl2 at ratios of 1:0.5, 1:1, 1:1.5, 1:2, 1:2.5, and 1:3 (HP0894:Zn^2+^, Supplementary Figure S3). A few residues in slow exchange mode and some residues in fast exchange mode were monitored. Most of the residues were saturated at 1:2 molar ratio (Figure 5), whereas the chemical shifts of a few residues in fast exchange mode continued to change, even after a 1:2 molar ratio, in a zinc ion concentration-dependent manner. These residues may have a low affinity for Zn^2+^ or may be located in a region remote from the Zn^2+^-binding core.

**Figure 6:**
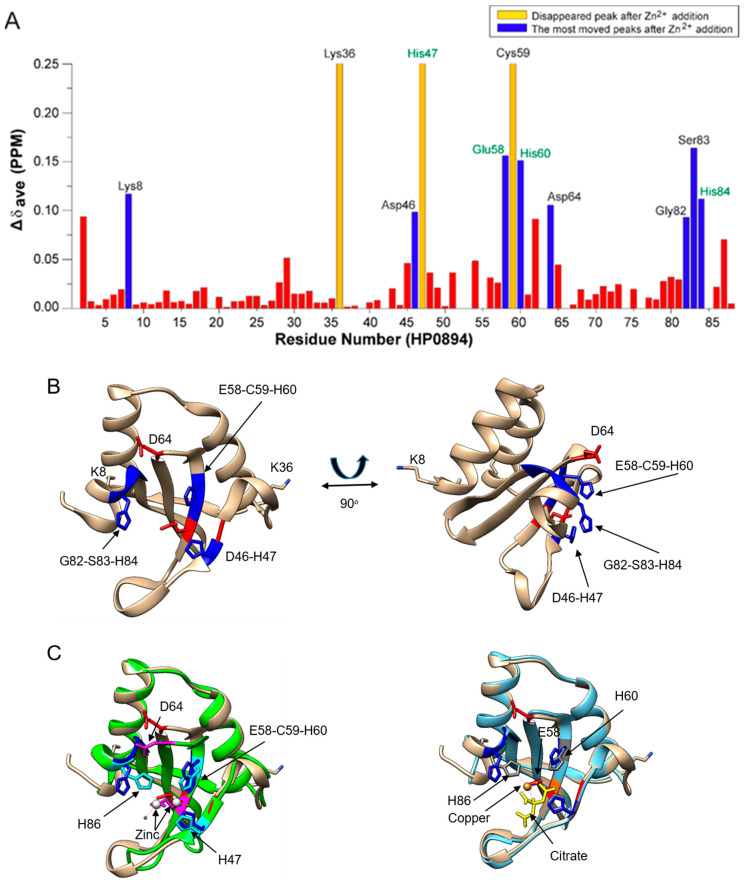
NMR perturbation of HP0894 upon addition of zinc ions. (**A**) A plot of the chemical shift changes upon addition of 300 μM zinc. The peaks that exhibited the most movement are illustrated in blue, and those that disappeared in yellow. The residues that showed mild shift are depicted by red bars. (**B**) The perturbed residues upon addition of zinc ions are mapped onto the structure to visualize their position in the three-dimensional structure of metal-bound HP0894. The image on the right is a 90° rotation about the y-axis of the image on the left. (**C**) Zinc-bound HP0892 (left. 4nrn) and copper-bound HP0894 (right, 4LSY) are compared with the apo-HP0894. The residues in pink and sky blue represent the residues of HP0892, while the residues in red and blue are the residues in the zinc binding site of HP0894. Two zinc ions form a complex with HP0892. The residues in grey correspond to the copper-coordinated residues of HP0894 (right panel). The salt, citrate, was also involved in copper coordination in the crystal structure.

The chemical shift perturbation (Δδavg, ppm) upon addition of ZnCl2 in 1:2 ratio was plotted along the amino acid sequence (Figure 6A). Rapid disappearances of Lys36, His47, and Cys59 were observed (yellow bars in Figure 6A), indicating that zinc binding affected the chemical environment of these residues. The most-shifted residues (blue bars in Figure 6A) were Lys8, Asp46, Glu58, His60, Asp64, Gly82, Ser83, and His84. These perturbed residues were mapped onto the HP0894 structure (PDB code: 4LTT) [14], as shown in Figure 6B. As observed in Figure 6B, most of these residues were placed on the central β-sheet of HP0894, and this region should be the binding pocket of Zn^2+^. The changes in Lys8 and Lys 38 were unexpected because these residues are located far from the binding pocket. Considering their positivity in the side chains, it is unlikely that they are directly involved in the interaction. Presumably, the significant changes in the two residues observed in the NMR spectrum may be indirectly derived from conformational changes in HP0894 upon binding to Zn^2+^.

Our previous report [20] evidenced that copper binds to HP0894 (PDB code 4LSY) through His47, Glu58, His60, and His84 residues, which is consistent with the current results. In addition, Asp64 which is conserved in HP0894, HP0892, and *E. coli* YafQ [20] (Asp64 in HP0894, Asp64 in HP0892, and Asp67 in *E. coli* YafQ), evidenced notable changes, whereas no change was identified in Asp64 of HP0892 [33,39].

## 4. Discussion

In this study, we evidenced that only Zn^2+^ had substantial effects compared to other metal ions and apo-HP0894. The RNase activity of HP0894 was reduced compared to that of apo-HP0894, indicating that Zn^2+^ had a significant inhibitory effect. Interestingly, none of the metal ions, including zinc, altered the secondary structure of HP0894 (Figure 2A). However, a significant increase in the Tm value was observed only when HP0894 was treated with Zn^2+^ (Figure 2B), suggesting that the folding state of HP0894 may become rigid upon Zn^2+^ binding. A change in the CD intensity in the near-UV region was observed with the addition of zinc, indicating that the solvent accessibility of the aromatic residues increased (Supplementary Figure S4). Based on NMR analysis, these aromatic residues appeared to be His47, His60, and His84. For instance, the binding of zinc to these residues may cause rearrangement of the side chains of aromatic residues, thereby exhibiting a change in the near-UV region. We previously reported another toxin in *H. pylori*, HP0892, which shares 53% sequence homology with HP0894 and binds to Zn^2+^ through three important His residues [39]. Glu58 of HP0892 was also suggested to be an important zinc coordinate residue, which is consistent with the current results. Similarly, HP0892 exhibited an increase in the Tm value due to Zn^2+^, while other divalent metal ions had a negligible effect on Tm. However, it was evidenced that two Zn^2+^ bound near β2 and β3 strands of HP0892; one zinc is coordinated with the residues His47, Glu58, and His60 of HP0892, and the other is coordinated with the residues Glu58 and His86 of HP0892 (Figure 6C). Thus, large differences between HP0892 and HP0894 exist in the zinc-binding stoichiometry and the coordinated pattern [33]. Our current study suggests that HP0894 may interact with a single zinc ion and that the four conserved residues, His47, Glu58, His60, and His84, form a catalytic core with zinc (Supplementary Figure S5). The chemical shift change of Asp64 was not observed in Asp64 of HP0892, which is another characteristic of HP0894.

The inhibitory role of zinc in RNA degradation with HP0894 was unexpected. It is well known that Zinc ions in bacteria are mainly used by metalloproteins with a total concentration in the 100 μM–1 mM range [40]. Zinc ions in cells are important for cell growth and development, where zinc is known to control gene transcription, growth, and differentiation. Furthermore, Zn^2+^ are involved in protein, nucleic acid, carbohydrate, and lipid metabolism [41]. Particularly, zinc in proteins is known for its catalytic, structural, and regulatory roles [42]. In metalloproteins, the binding of Zn^2+^ is known to occur at structural or catalytic sites, where these ions become an integral part of the protein [43], suggesting that zinc exhibits strong binding when a structural or catalytic role is associated. However, in the case of its regulatory or inhibitory roles, zinc regulation or inhibition causes phosphorylation, mitochondrial respiration, and neurotransmission [44]. Zinc often interacts with Asp, Glu, Cys, and His side chains, resulting in the inhibition of various enzymes [42]. Such species of inhibitory zinc sites hardly exhibit nanomolar to micromolar affinities but are predicted to be physiologically significant [45]. A similar inhibitory role of Zn^2+^ was observed in the human protein kallikrein 5 (hK5), where Zn^2+^ interacted with the side chain of His 57, resulting in the loss of enzymatic activity [46]. Protein tyrosine phosphatase (PTP) loses its phosphatase activity through zinc interactions, resulting in cell proliferation [41]. These results suggest that enzyme inhibition with Zn^2+^ such as HP0894 is more prevalent. In addition, it is imperative to explore in greater detail the effect of Zn^2+^ on the toxin–antitoxin mechanism to understand the biology of the toxin–antitoxin system.

Our previous research revealed that the HP0894 toxin has RNase activity, and the HP0894 toxin makes a stable complex with the HP0895 antitoxin, where the HP0894 toxin binds with the HP0895 antitoxin with nanomolar affinity [33]. HP0895 highly suppressed the RNase activity of HP0894, and its inhibitory ability was not affected by the presence of Zn^2+^ (Supplementary Figure S6). Under stressful conditions where the HP0895 antitoxin undergoes degradation by proteases, HP0894 may begin to degrade the produced RNAs to regulate protein production. In contrast, under unstressful conditions, HP0894 generally forms a very stable and tight complex with the antitoxin HP0895, resulting in HP0894 inhibition. During this process, there is presumably a time gap, as the new production of HP0895 may take some time. Finally, Zn^2+^ suppression of the RNase activity of HP0894 may have important implications in the quick response of bacteria; the time gap of toxin inhibition by antitoxins might be overcome by the abundance of Zn^2+^ in the bacteria. In conclusion, Zn^2+^ allows cells to quickly return to their growing state after a stressful situation has passed, acting as an antitoxin.

## Data Availability

Data are contained within the article.

## Supplementary Materials

The following supporting information can be downloaded at: https://www.mdpi.com/article/10.3390/life14020225/s1, The Supplementary Material contains six figures. Figure S1 shows HP0894 homologues from 13 species of *Helicobacter* are compared using the multiple sequence alignment program, CLUSTAL. The stars represent the conserved His residues that play a critical role in metal binding. The blue color represents the highly conserved residues between proteins. The sequence identities of 12 proteins compared to HP0894 were above 40%. Figure S2 shows sequence conservation of HP0894 among *Helicobacter plyori* strains. (A) BLASTP results showing 218 homologues of HP0894. (B) The multiple sequence alignment. The first ‘query_8782175′ is HP0894. The identical amino acids are represented by the symbol, dot. The color scheme depends on the amino acid types. Figure S3 shows the overlay of two-dimensional [1H-15N] HSQC spectra of 150 μM U-15N HP0894 alone (black) and upon the addition of a 1:0.5 molar ratio of ZnCl_2_ (lime green), 1:1 (orange), 1:1.5 (blue), and 1:2 (green). 1:2.5 ratio (purple), and 1:3 (red). The overlapping regions in the HSQC spectra are shown in the insets for clarity. The Figure S4 shows the positions of the aromatic residues upon the binding of zinc to HP0894. Figure S5 shows the binding regions of HP0894. The antitoxin protein, HP0895 binds mainly through the N-terminal helices (Leu2, Leu4–Asn20, Phe22–Glu38, and Leu40, green) while the Zn^2+^ ion binds the surface of β–sheet (red) that localizes opposite of the N-terminal helices. Figure S6 shows comparison of RNase activity of the HP0894 samples. Apo-HP0894 (black), 2 mM EDTA-added HP0894 (red), Zn^2+^-added HP0894 (blue), 20 μM (more than two equivalent of HP0894) HP0895-added HP0894 (green), and 2 mM Zn^2+^-HP0894-20 μM HP0895 (pink) are shown. The concentration of HP0894 was 8 μM. The inhibitory effect of the antitoxin for the RNase activity of HP0894 was very strong and did not show any significant difference depending on the presence of Zn^2+^.
