# Supplementary material for: Effect of Divalent Metal Ions on the Ribonuclease Activity of the Toxin Molecule HP0894 from Helicobacter pylori
Source: Life (Basel). 2024 Feb 5;14(2):225. doi: 10.3390/life14020225 (PMC10890551; doi:10.3390/life14020225)
Supplement: Supplementary file 1 [file life-14-00225-s001.zip › life-2819987-supplementary.pdf]

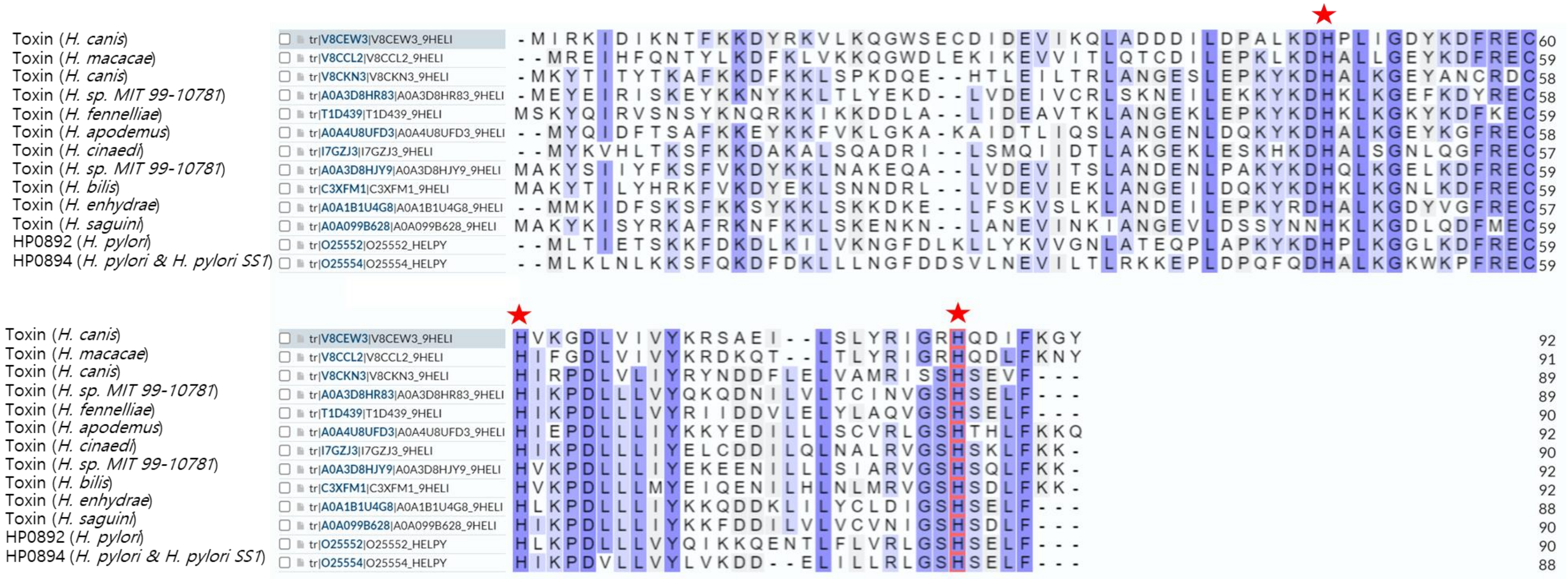

Supplementary Figure S1. HP0894 homologues from 13 species of Helicobacter are compared using the multiple sequence alignment program, CLUSTAL (Sievers F, Higgins DG. The Clustal Omega Multiple Alignment Package. Methods Mol Biol. 2021;2231:3-16). The stars represent the conserved His residues that play a critical role in metal binding. The blue color represents the highly conserved residues between proteins. The sequence identities of 12 proteins compared to HP0894 were above 40%.

(A)

Organism	Blast Name	Score	Number of Hits	Description
Helicobacter	e-proteobacteria		218	
. Helicobacter pylori	e-proteobacteria		200	
. . Helicobacter pylori 26695	e-proteobacteria	174	8	Helicobacter pylori 26695 hits
. . Helicobacter pylori Rif1	e-proteobacteria	173	1	Helicobacter pylori Rif1 hits
. . Helicobacter pylori Rif2	e-proteobacteria	173	1	Helicobacter pylori Rif2 hits
. . Helicobacter pylori 26695-1	e-proteobacteria	173	1	Helicobacter pylori 26695-1 hits
. . Helicobacter pylori Hp P-16	e-proteobacteria	171	1	Helicobacter pylori Hp P-16 hits
. . Helicobacter pylori Cuz20	e-proteobacteria	170	1	Helicobacter pylori Cuz20 hits
. . Helicobacter pylori Hp H-45	e-proteobacteria	170	2	Helicobacter pylori Hp H-45 hits
. . Helicobacter pylori HPAG1	e-proteobacteria	169	1	Helicobacter pylori HPAG1 hits
. . Helicobacter pylori Shi112	e-proteobacteria	168	1	Helicobacter pylori Shi112 hits
. . Helicobacter pylori B38	e-proteobacteria	167	1	Helicobacter pylori B38 hits
. Helicobacter pylori	e-proteobacteria	173	200	Helicobacter pylori hits

(B)

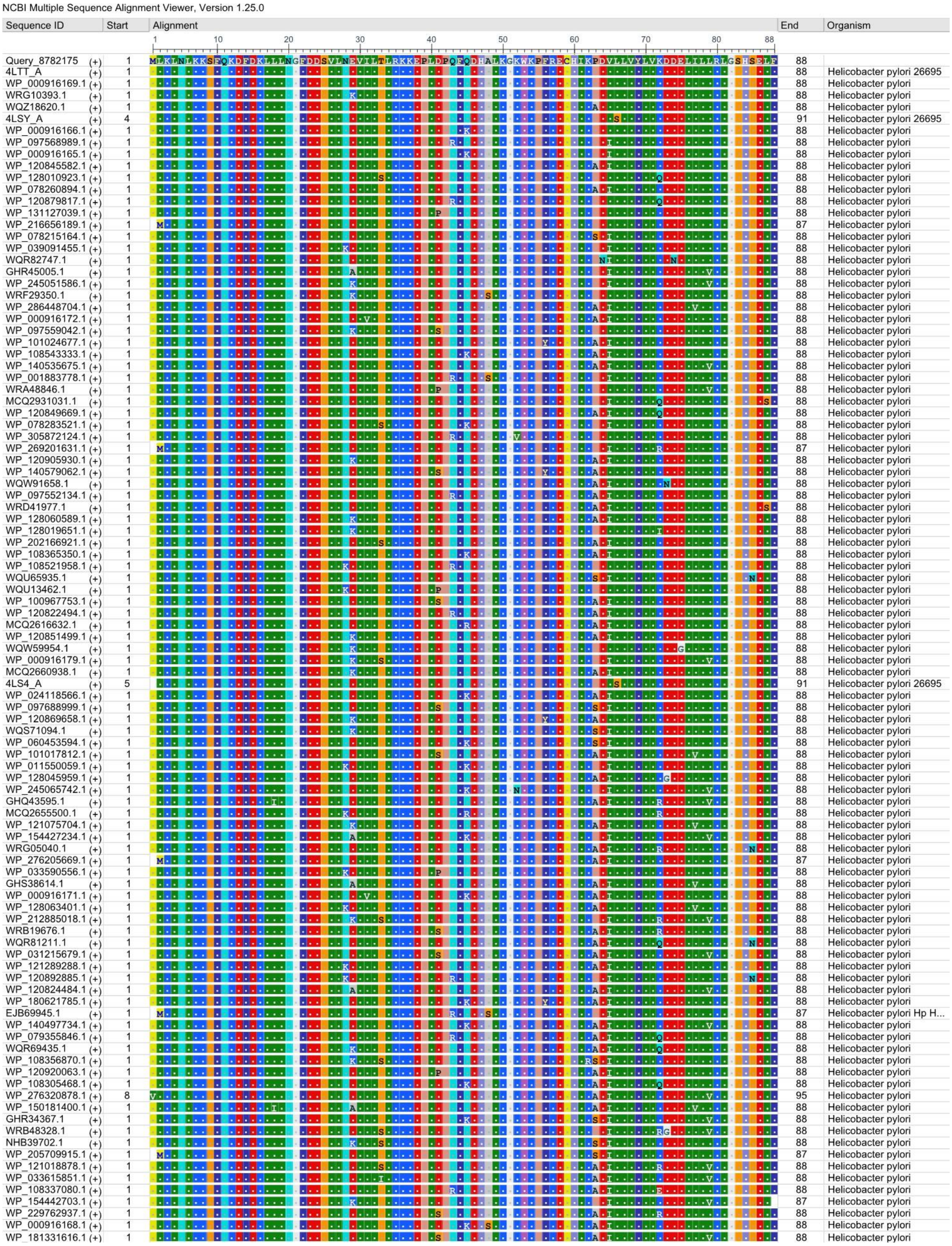

Supplementary Figure S2. Sequence homology of HP0894 among *Helicobacter plyori* strains. (A) Blast sequence alignment shows 218 homologues of HP0894. (B) The example of the sequence alignment. The first 'query_8782175' is HP0894. The conserved amino acids are represented by the symbol, dot. The color scheme depends on the amino acid types.

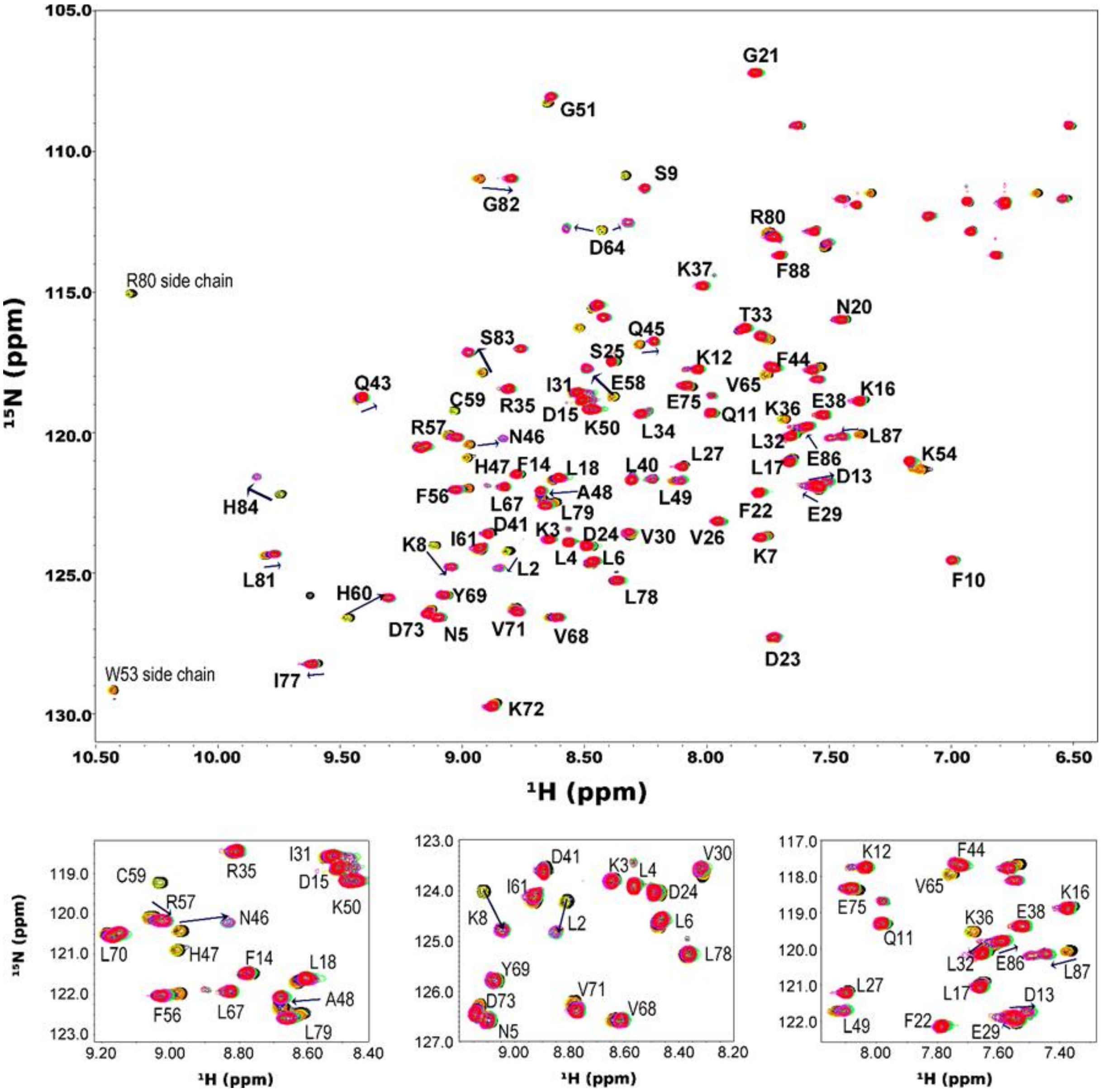

Supplementary Figure S3 NMR titration of HP0894 with increasing concentration of zinc ions. A series of two-dimensional [1H-15N] HSQC spectra of 150 μM U-15N HP0894 alone (black) and upon addition of 1:0.5 molar ratio of ZnCl2 (lime green), 1:1 ratio (orange), 1:1.5 ratio (blue), 1:2 (green). 1:2.5 ratio (purple), and 1:3 ratio (red) are illustrated. The overlapping regions in the HSQC spectra are illustrated in the inset for clarity, as well as the side chain of aromatic amino acids.

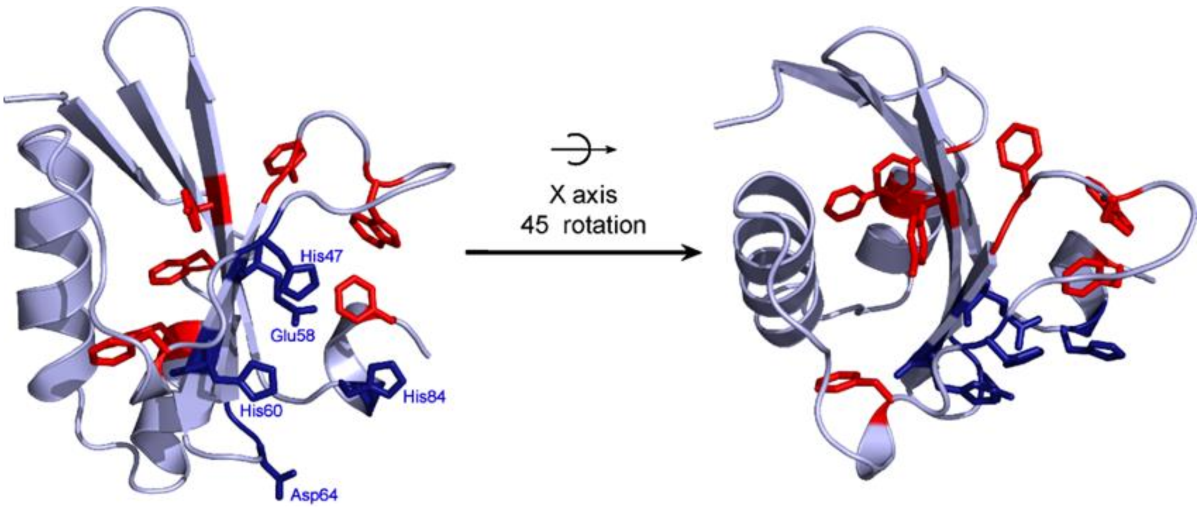

Supplementary Figure S4. Effect of zinc on aromatic residues of HP0894. The residues comprising the binding pocket of HP0894 is illustrated in blue. The effect of binding of zinc ions with HP0894 on aromatic residues as seen by near-UV region in circular dichroism is mapped into the structure of HP0894 and illustrated in red.

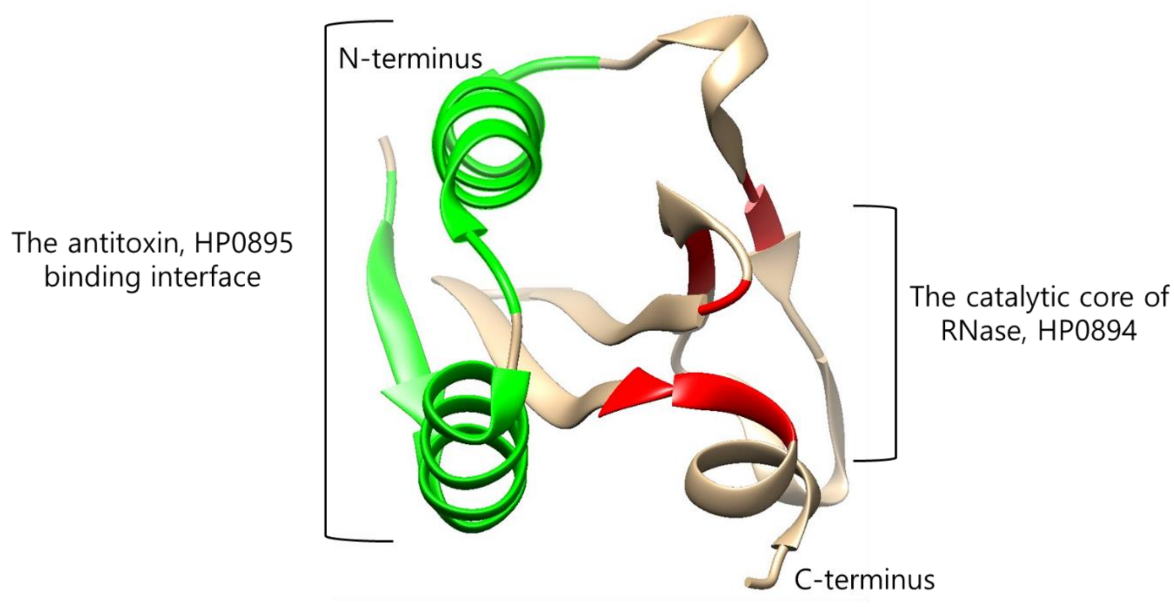

Supplementary Figure S5. The binding regions of HP0894. The antitoxin protein, HP0895 binds through mainly the N-terminal helices (Leu2, Leu4–Asn20, Phe22–Glu38, and Leu40, green) while the Zn^{2+} ion binds the surface of β–sheet (red) that localizes opposite of the N-terminal helices.

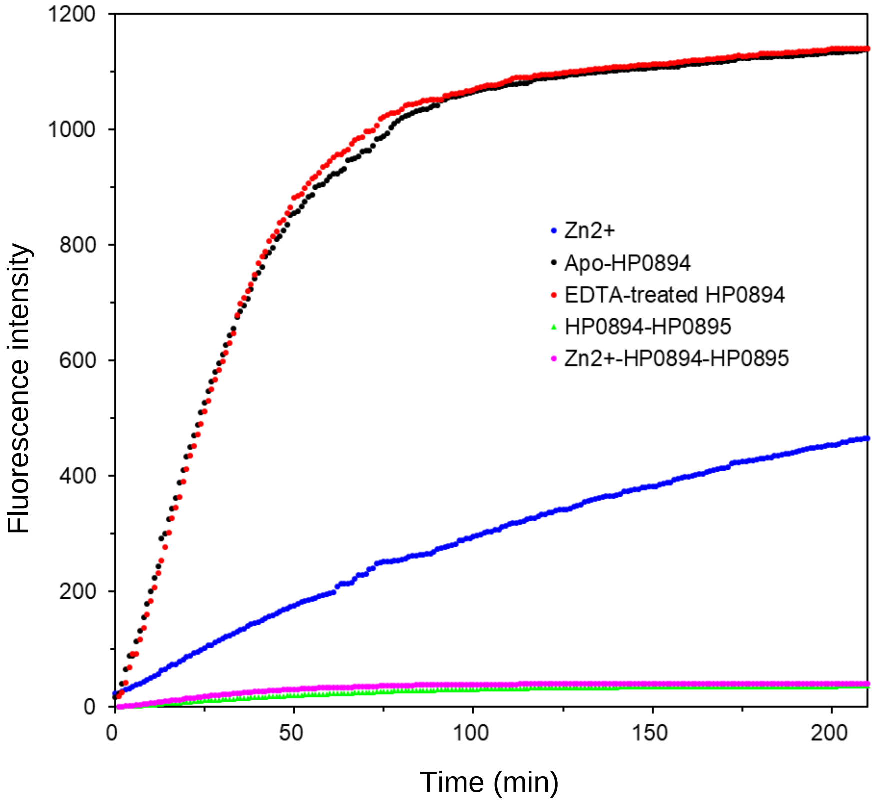

Supplementary Figure S6. Comparison of RNase activity of the HP0894 samples. Apo-HP0894 (black), 2 mM EDTA-added HP0894 (red), Zn^{2+}-added HP0894 (blue), 20 μM (more than two equivalent of HP0894) HP0895-added HP0894 (green), and 2 mM Zn^{2+}-HP0894-20 μM HP0895 (pink) are shown. The concentration of HP0894 was 8 μM. The inhibitory effect of the antitoxin for the RNase activity of HP0894 was very strong and did not show no significant difference depending on the presence of Zn^{2+}.
